# D-mannose promotes diabetic wound healing through inhibiting advanced glycation end products formation in keratinocytes

**DOI:** 10.1186/s10020-025-01070-3

**Published:** 2025-01-18

**Authors:** Jialiang Luo, Tianxing Wu, Jing Zhang, Zhicheng Liang, Weijie Shao, Di Wang, Lei Li, Daming Zuo, Jia Zhou

**Affiliations:** 1 Institute of Molecular Immunology, Key Laboratory of Infectious Diseases Research in South China, Ministry of Education, Guangdong Province Key Laboratory of Immune Regulation and Immunotherapy, School of Laboratory Medicine and Biotechnology, Southern Medical University, Guangzhou, 510515 Guangdong China; 2 Department of Immunology, School of Basic Medical Sciences, Southern Medical University, Guangzhou, 510515 Guangdong China; 3 Department of Dermatology, the Fifth Affiliated Hospital, Southern Medical University, Guangzhou, Guangdong China; 4 The Second School of Clinical Medicine, Zhujiang Hospital, Southern Medical University, Guangzhou, China; 5 Department of Dermatology, Dermatology Hospital of Southern Medical University, Southern Medical University, Guangzhou, China

**Keywords:** D-mannose, Diabetic wound healing, AGEs formation, Keratinocytes, AMPK/Nrf2/HO-1 signaling

## Abstract

**Background:**

Diabetic chronic foot ulcers pose a significant therapeutic challenge around the world, resulting in adverse effects and complications in patients. D-mannose is enriched in cirtus peel and exerts beneficial effects among various diseases, especially against inflammation-related disorders.

**Methods:**

Here, we examined the potential effect of D-mannose during wound healing process in streptozotocin (STZ)-induced diabetes mice in vivo and by culturing keratinocytes under high glucose condition in vitro. The skin lesion healing was recorded in photos and evaluated by histochemical staining. What’s more, the advanced glycation end products (AGEs) concentration in blood and mice skin was quantified. Apoptotic cells were assessed by flow cytometry and Western blotting. Inflammatory cytokines and cellular differential gene expression levels were measured by real-time PCR. The expression of the AMPK/Nrf2/HO-1 signaling-related molecules was determined by Western blotting.

**Results:**

We first found that topical supplementation of D-mannose remarkably improved skin wound healing in diabetes mice. Furthermore, both in vivo and in vitro experiments demonstrated that D-mannose reduced the AGEs generation. Mechanistically, D-mannose inhibited AGEs, then upregulated AMPK/Nrf2/HO-1 signaling in the context of high glucose to maintain keratinocyte normal functions, which positively influenced macrophage and fibroblast to accelerate diabetic wound healing. Noteworthily, these protective effects of D-mannose were abolished by the pretreatment with inhibitors of AGEs or AMPK.

**Conclusion:**

As far as we know, this is the first study exploring the protective role of D-mannose on diabetic wound healing via topical supplementation. We find that D-mannose protects keratinocytes from high glucose stimulation via inhibition of AGEs formation as well as orchestrates inflammatory microenvironment in diabetic wounded skin, suggesting its supplementation as a potential therapy to promote refractory wound healing in diabetic patients.

**Supplementary Information:**

The online version contains supplementary material available at 10.1186/s10020-025-01070-3.

## Introduction

Diabetes, as a critical global health issue, is a common metabolic disease affecting at least 425 million people worldwide and its prevalence surging continuously[1]. Patients suffering from diabetes are in a state of relentless inflammation, easily resulting in major clinical complications (especially diabetic foot ulcer) [2]. Approximately 25% of diabetic patients are plagued by chronic unhealing wounds and the risk of ulceration, infection, amputation and even death throughout their lifetime [3]. Generally speaking, the wound healing process is classified into four sequential phases: hemostasis, inflammation, proliferation and remodeling, involved with numerous cell populations and cytokines as well as signaling pathways within the skin microenvironment [4, 5]. Nevertheless, these four orderly stages are disorganized in diabetic wounds, where hyper-inflammatory response, infection and hypoxia lead to a constant inflammation stage instead of moving to remodeling. Up to now, there are numerous therapies against diabetic wounds in clinics, including blood glucose control, routine debridement, anti-infection therapy and surgical revascularization [6]. These present treatments are only suitable for mild-to-moderate diabetic wounds, without guarantee of no amputation and full recovery of skin function. Thereby, the introduction of effective drugs with biosafety is needed urgently to treat diabetic wounds and even reduce the risk of its progression.

Diabetic wound is a complicated pathological condition attributed to hyperglycaemia, advanced glycation end products (AGEs), reactive oxygen species (ROS) and inflammatory cytokines [7]. These unfavorable factors not only disrupt the physiological functions of skin cells but also cause further harmful cascades via cross-talk in the skin microenvironment. The high-glucose microenvironment is pivotal for the occurrence and progression of diabetic wounds, in which glucose is conjugated to macromolecules thus generating AGEs [8, 9]. Accumulating evidence revealed that AGEs aggravate chronic inflammation and oxidative stress to advance disease progression [10]. Recently, the impaired function of keratinocyte has been confirmed as a substantial cause delaying diabetic wound healing process [11, 12]. As the predominant cell population in epidermis, keratinocytes are responsible for the major source of AGEs formation as well as compromised wound healing in diabetics [13]. AGEs could interfere with the normal functions of keratinocytes, including cell viability, migration and differentiation and inflammatory cytokines production [14–16]. In addition, inflammatory keratinocytes induced by AGEs or high glucose would further influence other neighbouring skin cell populations [17, 18] to develop nonhealing wounds in the context of hyperglycemic condition.

D-mannose is a natural monosaccharide widely enriched in citrus, cranberry and detectable in human plasma [19]. D-mannose is known for its potent anti-inflammatory function among multiple diseases through downregulating pro-inflammatory macrophages and effector T cells, while upregulating anti-inflammatory gut microbiome and regulatory T cells [20]. Supplementation of D-mannose has been proved as a beneficial therapeutic treatment for people suffering from recurring urinary tract infections as well as carbohydrate-deficient glycoprotein syndrome patients in clinical practice [21, 22]. Our lab previously found the beneficial supplementation of D-mannose alleviates numerous inflammatory diseases, including alcoholic liver disease and colitis [23, 24]. Moreover, we first reported that D-mannose could significantly inhibit abnormal inflammatory response to ameliorate atopic dermatitis via skin topical application [25]. However, the potential effect and the molecular mechanism of D-mannose during wound healing under high glucose are still inconclusive.

In this study, D-mannose was applied to wounds on diabetic mice induced by streptozotocin (STZ) via skin topical supplement. For in vitro experiments, keratinocytes were incubated with D-mannose under high glucose condition, aiming at unveiling the protective role and underlying mechanism of D-mannose during diabetic wound healing. Overall, our present results demonstrate that D-mannose could disturb glucose metabolism in keratinocytes to accelerate diabetic wound healing. Mechanistically, D-mannose inhibits AGEs production, and subsequently upregulates AMPK/Nrf2/HO-1 signaling pathway to maintain the physiological functions in keratinocytes. These data together indicate D-mannose topical supplementation is a promising clinical treatment for refractory wound healing in diabetic patients.

## Methods

### Experimental animals

Male Balb/c (6–7 weeks old) were purchased from the Experimental Animal Center of Southern Medical University. The animals were maintained under a 12-h light/dark cycle in a specific pathogen-free animal facility at a controlled temperature (20–25 °C) and humidity (50 ± 5%). All animal experiments in this study were approved by the Welfare and Ethical Committee for Experimental Animal Care of Southern Medical University.

For in vivo experiments, all mice were randomized into four groups: control, D-mannose, streptozotocin (STZ), STZ with D-mannose treatment. Mice in STZ and STZ combined with D-mannose groups were intraperitoneally injected with STZ (50 mg/kg i.p.) every day for 5 consecutive days. The establishment of mice diabetes by STZ injection was according to a previous study [26]. Since STZ could induce fatal hypoglycemia, mice were supplied with 5% glucose in water to prevent hypoglycemia during the 5 days of STZ administration [27]. After 1 week, blood glucose was examined and mice with blood glucose level > 300 mg/dl were subjected to further experiments. A full-thickness wound was excised on the back of the mice on day 0. D-mannose (3% w/v, dissolved in PBS, phosphate buffered saline) was topically applied to mice in D-mannose group and STZ with D-mannose group on dorsal skin (100 µL) every day to the mice. The dosage of 3% D-mannose supplementation on skin was chosen on the basis of our lab’s preliminary experiments [25]. Meanwhile, the control and STZ group mice received the same volume of PBS. The wound healing process as well as blood glucose was recorded every day. Mice were anaesthetised with pentobarbital sodium and sacrificed on day 13.

### Histochemistry and immunofluorescence

Paraffin-embedded, formalin-fixed 5-µm-thick tissue sections or cells grown on coverslips in 12-well plate were processed for staining with the staining dye or primary antibodies. The following procedures were performed as we previously described [28]. Images were obtained by laser scanning microscope system (Nikon Eclipse Ni, Japan).

### Cell culture

The human keratinocyte NHEK or HaCaT cells were both grown and maintained in low glucose Dulbecco’s modified Eagle’s medium (DMEM, low glucose) supplemented with 10% heat-inactivated fetal bovine serum in a humidified chamber with 5% CO_2_ at 37 °C. NHEK or HaCaT were treated with D-mannose (5 mM) and high glucose (10, 25, 50 mM) or AGEs (50, 100 µg/mL) for 24–48 h. In some experiments, NHEK were treated with Compound C (10 µM) or ALT711 (10 µM) 2 h ahead of indicated stimulation.

In some experiments, THP-1 (human monocytic leukemia cell line) and HFF-1 (human skin fibroblast cell line) were used.

### Reagents and antibodies

D-mannose (M2069) was obtained from Sigma-Aldrich (USA). STZ (S1312) and Compound C (S7840) were bought from Selleck Chemicals (USA). ALT711 (HY-106024B) and glucose (HY-B0389) were purchased from MCE (China). For Western blotting and immunofluorescence, the primary antibodies used to detect proteins were described as follows: Antibodies against AMPK (2523), phosphor-AMPK (2535), Nrf2 (12721), HO-1 (43966), Bax (2772) and Bcl-2 (15071) were from Cell Signaling Technology (USA). Anti-β-actin antibody (81115-1-RR) was purchased from Proteintech (China). AGEs antibody (bs-1158R) and AGEs (bs-1158P) were from Bioss (China).

### AGEs measurement

AGEs measurement in cells and mice skin followed the manufacturer’s instructions (Abcam, ab238539) [8]. In short, both cells with indicated treatment and mice skin tissues were lysed and the supernatant was collected, then AGEs concentration was assayed with a microplate reader.

### Cell viability assay

NHEKs cells (3 × 10^3^ cells per well) were seeded into 96-well plates and treated with D-mannose and glucose for 48 h, followed by incubating with CCK-8 solution (Beyotime Biotechnology) for 4 h. Cell viability was detected by microplate reader at an absorbance wavelength at 450 nm.

### Cell scratch assay

NHEK cells (8 × 10^5^ cells per well) were seeded into 6-well plates. Upon nearly 100% cell confluence, a sterile pipette was used to scratch straight lines on cells, followed by medium washing to remove the detached cells. Subsequently, the cells were cultured with the indicated treatment (high glucose, AGE with or without mannose treatment) and photographs of the scratch wound in different samples were recorded at 0 and 48 h.

### Flow cytometry

The apoptotic cells of NHEK were measured by flow cytometry. Briefly, NHEK with indicated treatments were collected and stained with PI and Annexin-V for apoptosis assay., then were subjected to flow cytometry detection.

In some experiments, the expression of CD86 inTHP-1 after M1 polarization was measured by flow cytometry. After indicated treatment, THP-1 was collected and stained with CD86 antibody for 30 min in dark, then was subjected to flow cytometry detection.

Data were acquired on LSRII/Fortessa flow cytometer (BD Biosciences, Germany) and analyzed using FlowJo software.

### RNA isolation and quantitative real-time PCR

Total RNA is isolated from dorsal skin by using TRIzol (TransGen Biotech, China) according to the manufacturer’s instruction. Further experiment procedures were performed as we previously described [25]. The expression levels of target genes were normalized with respect to β-actin gene expression and the primers were shown in the supplementary Tables 1 and 2.

### Immunoblotting

NHEK cells or dorsal skin were lysed in cell lysis buffer for Western blotting (Beyotime Biotechnology, China) containing PMSF (Beyotime Biotechnology) on ice for 30 min, centrifuged at 14,000 g for 15 min at 4 °C, and the supernatants were collected. After gel electrophoresis, the blot was subjected to blocking and incubation of primary antibody at 4 °C overnight, followed by secondary antibody incubation at room temperature for 1 h.

### The culture and treatment of THP-1 and HFF-1

The culture medium of NHEK containing glucose and D-mannose was removed and replaced with new culture medium. After 24 h, the new culture medium was then centrifuged and supernatants were collected. THP-1 treated with PMA plus LPS (Selleck) for M1 polarization as previously described [29] or HFF-1 were cultured with 30% NHEK-culture medium for 48 h and collected for further analysis.

### Statistical analysis

All values were expressed as mean ± SEM. One-way ANOVA followed by Tukey’s post-hoc tests was used for multiple group comparisons. *P* < 0.05 was considered significant. Statistics were calculated with GraphPad Prism version 8, GraphPad Software.

## Results

### D-mannose promoted diabetic wound healing in STZ-induced diabetic mice

**Fig. 1:**
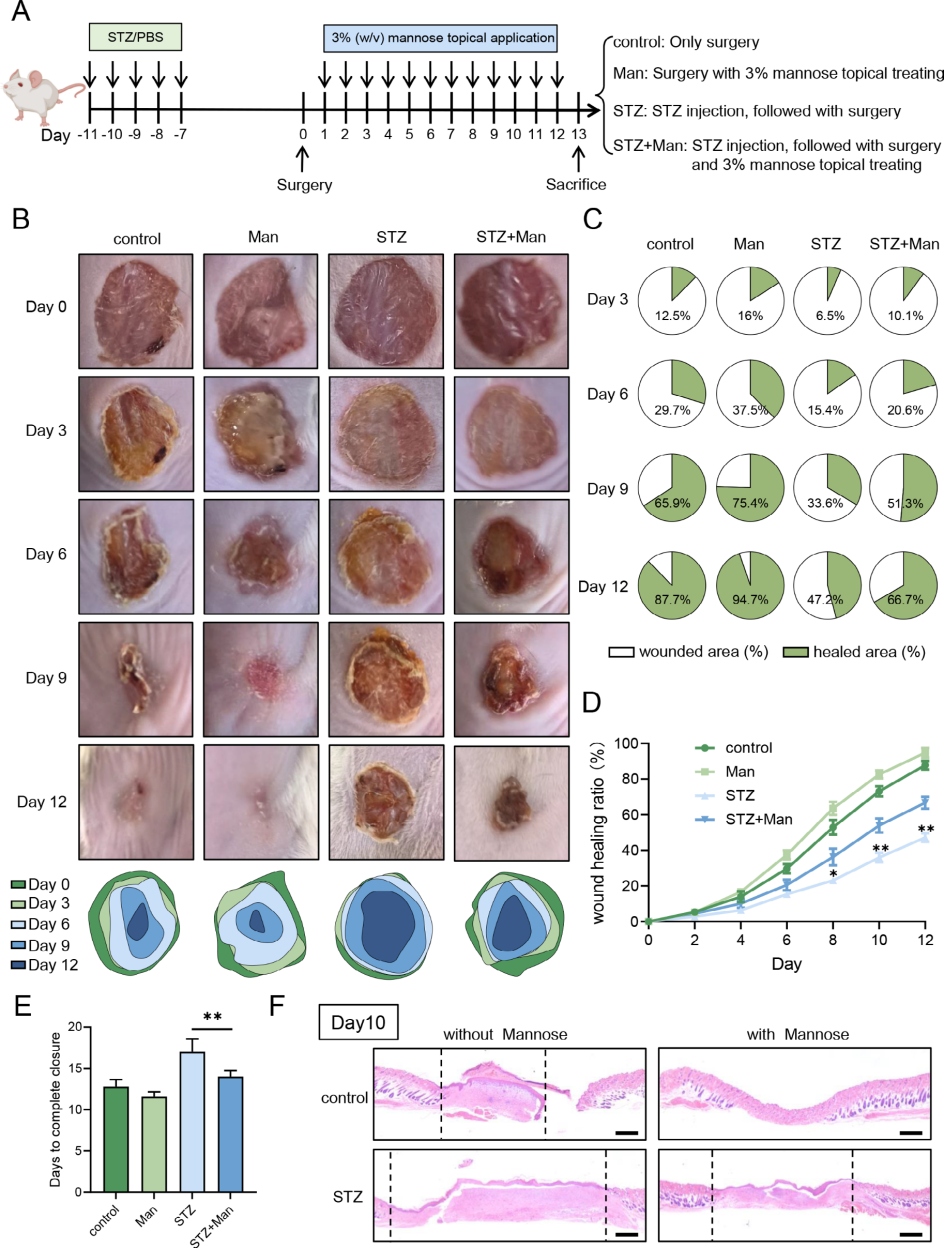
D-mannose promoted diabetic wound healing in STZ-induced mice. Balb/c mice (*n* = 5) received STZ injection and wound punch onto dorsal skin to establish diabetic wound healing, along with or without D-mannose topical application, shown as the scheme (**A**). (**B**) Representative photomicrographs of wounds on mice skin and traces of wound closure. (**C**) The wound healing ratio was calculated every other day during the wound healing process. (**D**) Fractions of wounds healed by different treatments on day 3, 6, 9 and 12. (**E**) Summary of the complete wound-closure times. (**F**) Representative photomicrographs of H&E staining in the skin lesional tissues. Wound lesion size is indicated between the dashed line. Scale bars = 200 μm. Values are expressed as the mean ± standard error; *P < 0.05, **P < 0.01. One-way ANOVA followed by Tukey’s post-hoc tests for multiple group comparisons

To explore the role of D-mannose supplementation on diabetic wound healing, Balb/c mice received STZ injection to establish diabetes model, followed by wound excision and skin topical administration of 3% D-mannose according to our previous study [25] (Fig. 1A). Wound healing process on diabetic mice were greatly slower than mice without STZ induction (Fig. 1B). Moreover, it is noted that D-mannose topical supplement significantly accelerated diabetic wound healing, while slight improvement in non-diabetic mice (Fig. 1B). Apart from images of wounds, the wound closure ratio and wounded area fraction both confirmed that D-mannose administration promoted wound healing in diabetic mice (Fig. 1C, D). Consistently, skin topical application of D-mannose reduced the time of complete wound healing (Fig. 1E), which is also supported by H&E staining (Fig. 1F). To sum up, skin topical supplement of D-mannose could accelerate wound healing in diabetic mice.

### D-mannose inhibited AGEs production

**Fig. 2:**
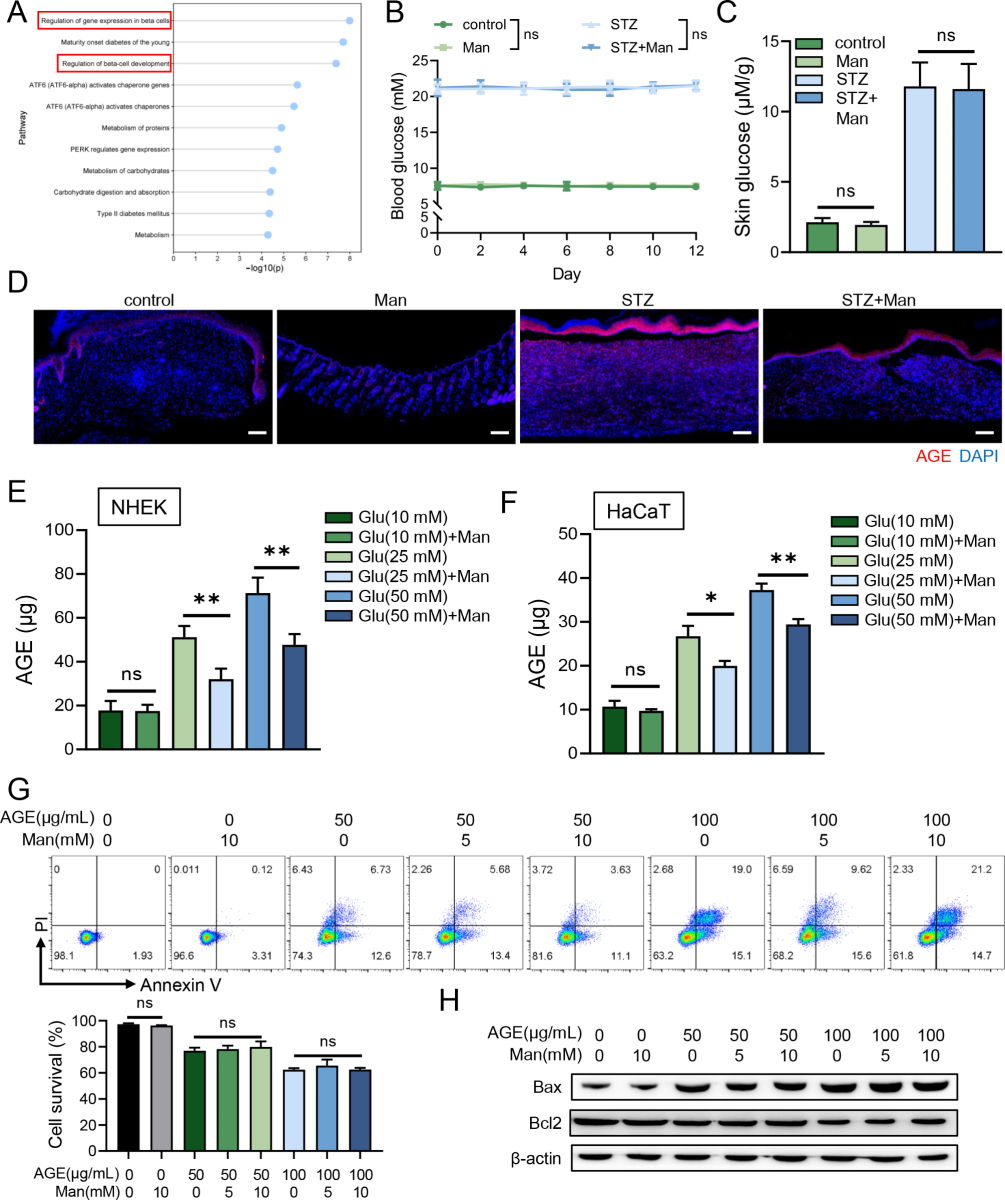
D-mannose inhibited AGEs production. (**A**) By the utilization of CTD database, the pathways are enriched significantly among genes interacting with D-mannose. (**B**) The blood glucose concentration was recorded during the treatment process. (**C)** The glucose concentration on mice lesional skin on day 13. (**D**) Representative photomicrographs of AGEs (red) in mice skin by immunofluorescence analysis. Scale bars = 100 μm. The content of AGEs in HaCaT (**E**) and NHEK cells (**F**) treated with high-glucose and D-mannose. (**G**) The apoptosis cells on the treated NHEK with flow cytometry analysis. (**H**) The protein level of apoptosis-related protein (Bax, Bcl2) was assessed by western blot. Values are expressed as the mean ± standard error of the mean from three independent experiments; ns, not significant, *P < 0.05, **P < 0.01. One-way ANOVA followed by Tukey’s post-hoc tests for multiple group comparisons

By the utilization of a public database (https://ctdbase.org), mannose was found could regulate beta cells which are cruicial to regulate blood glucose (Fig. 2A), so we first hypothesized that topical D-mannose supplement could directly reduce blood glucose concentration thus promoting diabetic wound healing. The concentration of blood glucose from different time points as well as skin glucose in mice was recorded, however, D-mannose did not alter glucose levels at all (Fig. 2B, C). Previous studies have demonstrated D-mannose could disturb glucose metabolism in tumor cells [30]. As high glucose contributed to AGEs transformation, which greatly interfered with diabetic wound healing [9, 10], we next assessed the AGEs levels on mice skin and found that D-mannose indeed greatly reduced AGEs levels in diabetic mice skin, epidermis in particular (Fig. 2D). As keratinocytes predominate in epidermis and are an important source of AGEs [13], two keratinocyte cell lines (HaCaT and NHEK) were used and incubated with glucose in different concentrations to examine the inhibitory effect of D-mannose on AGEs production. The results showed that D-mannose significantly suppressed AGEs production in both cell lines under the hyperglycemia condition (Fig. 2E, F). Furthermore, we stimulated NHEK with exogenous AGEs and D-mannose and found that D-mannose could not influence cell survival ratio (Fig. 2G) and influence apoptosis-related protein expression (Fig. 2H) with AGEs addition. Taken together, D-mannose promoted wound healing under hyperglycemia condition via suppressing AGEs production.

### D-mannose maintained physiologic functions of keratinocytes under high-glucose condition

**Fig. 3:**
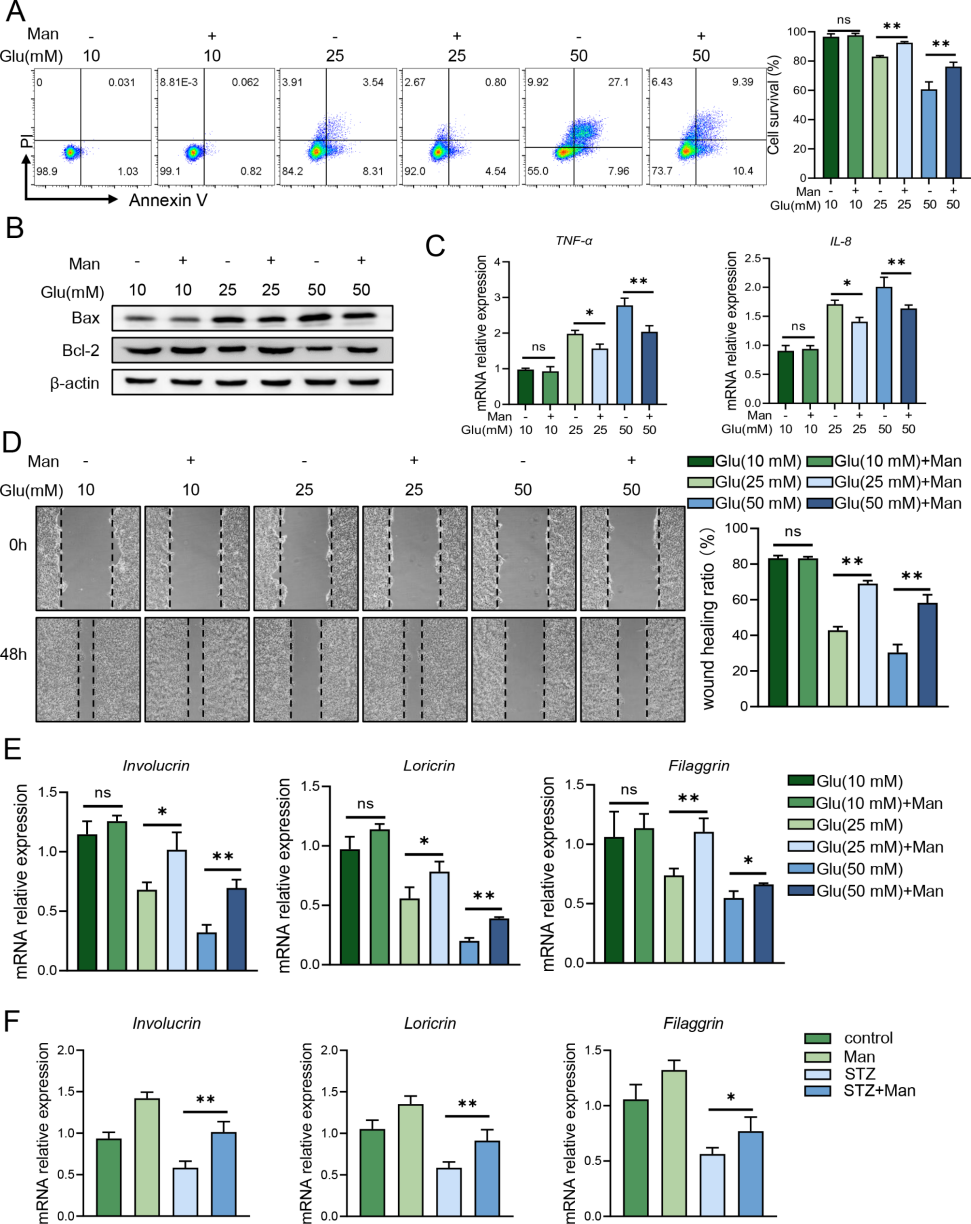
D-mannose maintained physiologic functions of keratinocytes under high-glucose condition. NHEK cells were stimulated with different concentrations of glucose and/or D-mannose for 24–48 h. (**A**) The apoptosis cells on the treated NHEK with flow cytometry analysis. (**B**) The protein level of apoptosis-related protein (Bax, Bcl2) was assessed by western blot. (**C**) The mRNA level of TNF-α and IL-8 in NHEK was evaluated by quantitative RT-PCR analysis. (**D**) The ability of migration was examined in treated NHEK by cell scratching assay. (**E**) The mRNA level of differentiation-related genes in NHEK was evaluated by quantitative RT-PCR analysis. (**F**) The mRNA level of differentiation-related genes in mice skins was evaluated by quantitative RT-PCR analysis. Values are expressed as the mean ± standard error of the mean from three independent experiments; ns, not significant, *P < 0.05, **P < 0.01. One-way ANOVA followed by Tukey’s post-hoc tests for multiple group comparisons

The functions of epidermal keratinocytes are impaired in high-glucose environments, exhibiting excessive inflammatory cytokines production, increased cell apoptosis, inhibited migration ability, down-regulated differentiation and so forth [8, 31]. To further elucidate the beneficial role of D-mannose on keratinocytes during the diabetic wound healing process, cell survival (Fig. 3A) and apoptosis-related protein expression were evaluated and the results indicated D-mannose protected NHEK from apoptosis (Fig. 3B). We then examined the mRNA expression of Tumor necrosis factor-α (TNF-α) and Interleukin-8 (IL-8) in NHEK (Fig. 3C) and found that D-mannose was able to potently inhibit inflammatory cytokines production in keratinocytes under high-glucose condition. Besides, cell scratch assay exhibited D-mannose improves cell migration (Fig. 3D). Keratinocyte-differential mRNA genes (Involucrin, Loricrin and Filaggrin) expression in cell lines (Fig. 3E) and mice skin (Fig. 3F) both confirmed D-mannose recovered normal differentiation in the context of hyperglycimia. In summary, D-mannose improved the physiologic functions of keratinocytes in high glucose environment.

### D-mannose-mediated keratinocyte protection contributed to decreased M1 polarization and fibroblast activation during diabetic wound healing

**Fig. 4:**
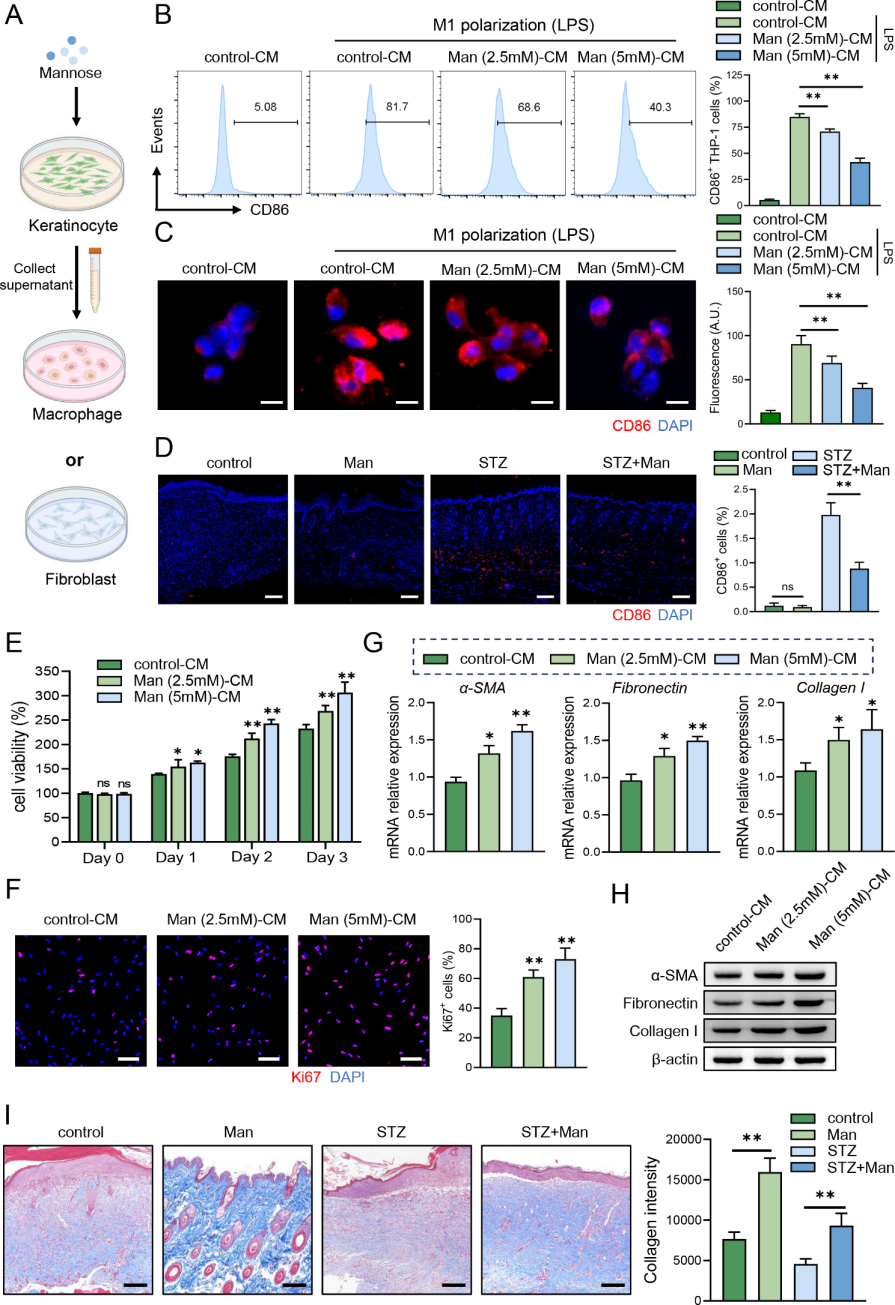
D-mannose-mediated keratinocyte protection contributed to decreased M1 polarization and fibroblast activation during diabetic wound healing. (**A**) Schematic representation of D-mannose-cultured medium of NHEK being collected and added to THP-1 or HFF-1 cells. (**B**) The percentage of CD86^+^ cells by flow cytometry. (**C**) Representative photomicrographs of CD86 (red) in THP-1 cells by immunofluorescence analysis. Scale bars = 10 μm. (**D**) Representative photomicrographs of CD86 (red) in mice lesional skin by immunofluorescence analysis. Scale bars = 100 μm. (**E**) Cell viability was determined by CCK-8 assay in NHEK. (**F**) Representative photomicrographs of Ki67 (red) in HFF-1 cells by immunofluorescence analysis. Scale bars = 50 μm. (**G**) The mRNA level of α-SMA, fibronectin and Collagen I in NHEK was evaluated by quantitative RT-PCR analysis. (**H**) The protein level of α-SMA, Collagen I and fibronectin in NHEK was assessed by western blot. (**I**) Representative photomicrographs of Masson’s trichrome staining in the mice skin tissue. Scale bars = 100 μm. Values are expressed as the mean ± standard error of the mean from three independent experiments; ns, not significant, *P < 0.05, **P < 0.01. One-way ANOVA followed by Tukey’s post-hoc tests for multiple group comparisons

Upon the stimulation of high glucose, keratinocytes could release inflammatory cytokines to influence other cell populations in the skin microenvironment [32], therefore hindering the wound healing process in diabetic patients. Since D-mannose could downregulate inflammatory cytokines in keratinocytes, the supernatant of D-mannose-treated keratinocytes was collected and added to macrophages or fibroblasts for the indicated time (Fig. 4A). On the one hand, we observed that D-mannose-cultured medium (mannose-CM) could significantly inhibit the frequency of CD86^+^ THP-1 cells during M1 polarization (Fig. 4B). Cellular immunofluorescence further exhibited supernatant from D-mannose-treated keratinocytes suppressed M1 polarization, especially that with higher concentration of D-mannose (Fig. 4C). In line with in vitro experiments, D-mannose treatment greatly reduced the numbers of CD86^+^ macrophages in mice lesional skin compared that with only STZ-induced mice (Fig. 4D). On the other hand, we examined cell proliferation ability of fibroblasts (Fig. 4E, F) and found supernatant from D-mannose-treated keratinocytes increased HFF-1 proliferation in a dose-dependent manner. What’s more, the collagen deposition of fibroblasts is impaired during high glucose environment but D-mannose treatment greatly increased collagen production both in vitro (Fig. 4G, H) and in vivo (Fig. 4I). Taken together, D-mannose could orchestrate macrophages and fibroblasts through keratinocytes, which together improves diabetic wound healing.

### D-mannose protected keratinocytes via AMPK/Nrf2/HO-1 signaling pathway

**Fig. 5:**
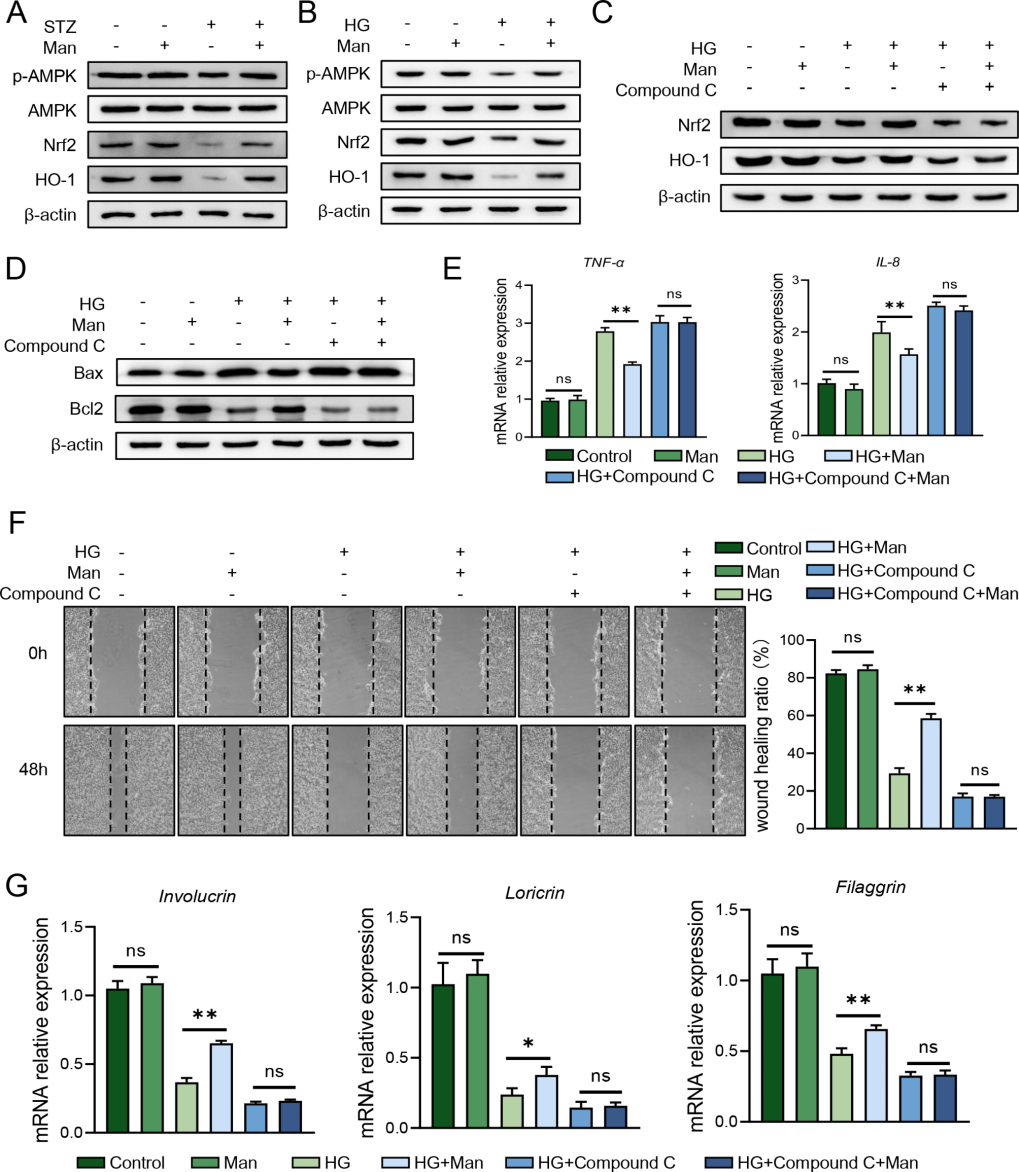
D-mannose protected keratinocytes via AMPK/Nrf2/HO-1 signaling pathway. (**A**) The protein level of AMPK, p-AMPK, Nrf2, HO-1 in skin tissues of mice was analyzed by western blotting. NHEK cells were stimulated with high glucose and/or D-mannose for 24–48 h. (**B**) The protein level of AMPK, p-AMPK, Nrf2, HO-1 in NHEK was analyzed by western blotting. NHEK cells were pre-treated with Compound C for 2 h ahead of treatment with high glucose and D-mannose. (**C**) The protein level of Nrf2, HO-1 in NHEK was analyzed by western blotting. (**D**) The protein level of apoptosis-related protein (Bax, Bcl2) was assessed by western blot. (**E**) The ability of migration was examined in treated NHEK by cell scratching assay. (**F**) The mRNA level of TNF-α and IL-8 in NHEK was evaluated by quantitative RT-PCR analysis. (**G**) The mRNA level of differentiation-related genes in NHEK was evaluated by quantitative RT-PCR analysis. Values are expressed as the mean ± standard error of the mean from three independent experiments; ns, not significant, *P < 0.05, **P < 0.01. One-way ANOVA followed by Tukey’s post-hoc tests for multiple group comparisons

Emerging evidence showed that biological function of D-mannose is dependent on AMPK signaling [33, 34]. Moreover, AMPK/Nrf2/HO-1 not only protects cells from high glucose condition [35] but also acts as a major pathway compromised by excessive AGEs [36, 37]. Thus, we assessed this pathway in mice skin and found that D-mannose indeed upregulated AMPK/Nrf2/HO-1 signaling pathway (Fig. 5A), which is also confirmed in keratinocytes stimulated with high glucose (Fig. 5B). To further confirm D-mannose protective role in keratinocytes via AMPK, we applied Compound C, a potent selective AMPK inhibitor, on NHEK before high glucose and D-mannose stimulation. We saw that the upregulation of Nrf2/HO-1 signaling pathway was significantly inhibited by Compound C and the differences between D-mannose treated or untreated cells were abolished (Fig. 5C). What’s more, the apoptosis-related protein expression (Fig. 5D), cell migration (Fig. 5E), mRNA levels of pro-inflammatory TNF-α, IL-8 (Fig. 5F), and the expression of keratinocyte differentiation-related genes (Fig. 5G) between D-mannose-treated and untreated cells became comparable. Overall, D-mannose protected the physiologic functions of high-glucose-stimulated keratinocytes through AMPK/Nrf2/HO-1 pathway.

### D-mannose inhibited AGEs generation to upregulate AMPK/Nrf2/HO-1 pathway in high-glucose-stimulated keratinocytes

**Fig. 6:**
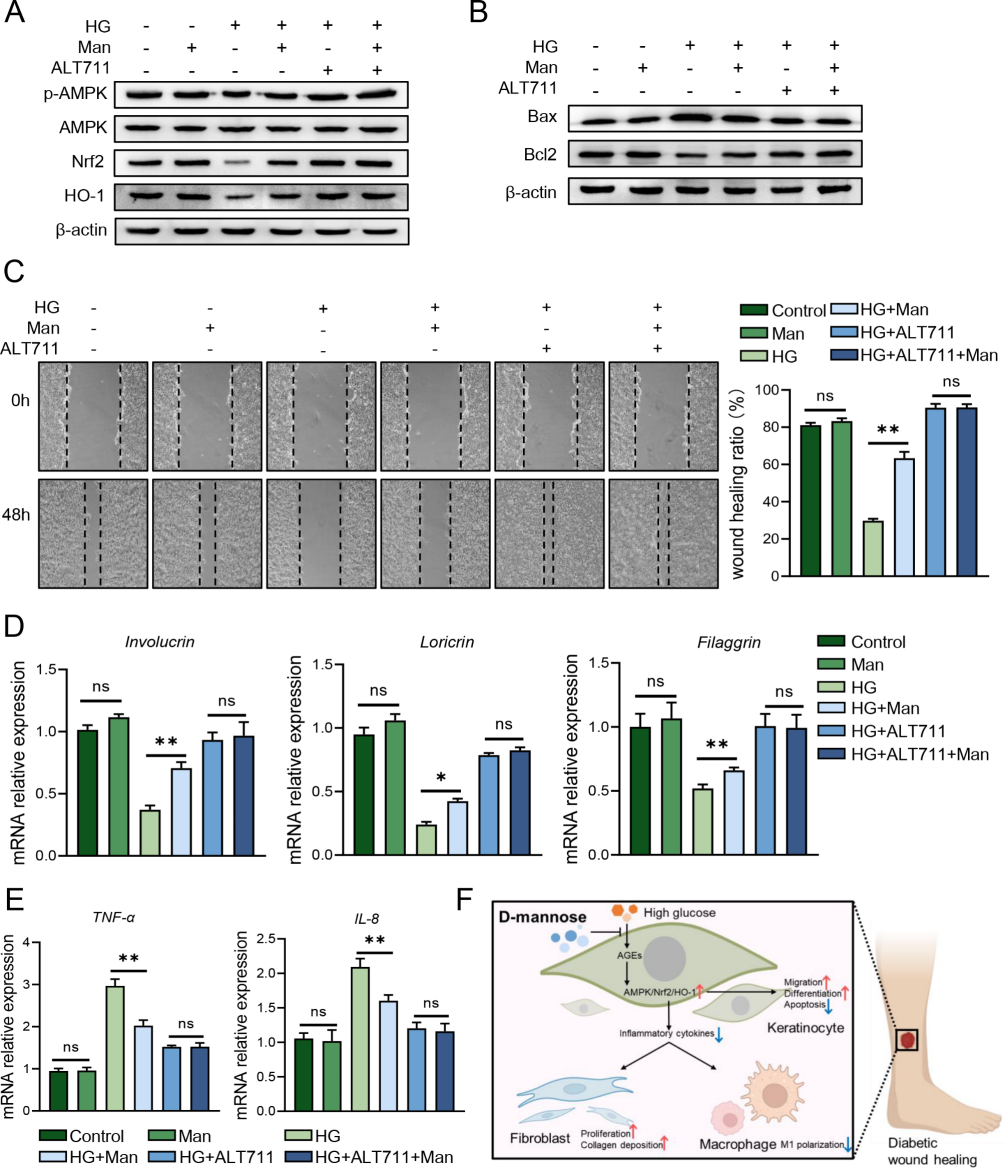
D-mannose inhibited AGEs generation to upregulate AMPK/Nrf2/HO-1 pathway in high-glucose-stimulated keratinocytes. NHEK cells were pre-treated with ALT711 for 2 h ahead of treatment with high glucose and D-mannose. (**A**) The protein level of AMPK, p-AMPK, Nrf2, HO-1 in NHEK was analyzed by western blotting. (**B**) The protein level of apoptosis-related protein (Bax, Bcl2) was assessed by western blot. (**C**) The ability of migration was examined in treated NHEK by cell scratching assay. (**D**) The mRNA levels of differentiation-related genes in NHEK were evaluated by quantitative RT-PCR analysis. (**E**) The mRNA level of TNF-α and IL-8 in NHEK was evaluated by quantitative RT-PCR analysis. (**F**) Schematic representation of the proposed mechanism for D-mannose in STZ-induced diabetic wound healing. Values are expressed as the mean ± standard error of the mean from three independent experiments; ns, not significant, *P < 0.05, **P < 0.01. One-way ANOVA followed by Tukey’s post-hoc tests for multiple group comparisons

The above experimental results have demonstrated that D-mannose suppressed AGEs generation and boosted up AMPK/Nrf2/HO-1 pathway, but the underlying relationship remains unclear. Here, AGEs generation specific inhibitor (ALT711) was applied to NHEK cells before glucose and D-mannose treatment. We found that the phosphorylation of AMPK was significantly inhibited by inhibitor ALT711, and the expression of Nrf2 and HO-1 was suppressed as well (Fig. 6A). This pretreatment of ALT711 could eliminate the differences between D-mannose treated or untreated high-glucose-induced NHEK, as determined by comparable apoptosis-related protein expression (Fig. 6B), cell migration (Fig. 6C), differentiation-related genes expression (Fig. 6D) and mRNA levels of pro-inflammatory TNF-α, IL-8 (Fig. 6E). These results together suggested that D-mannose achieved its diabetic wound healing function through suppressing AGEs generation which could upregulate downstream AMPK/Nrf2/HO-1 pathway in keratinocytes.

## Discussion

D-mannose is a kind of monosaccharide widely distributed in natural plants, being well known as a beneficial supplement in treating numerous diseases [19]. Refractory diabetic wound healing is a serious complication of diabetes, leading to limb amputation and even morbidity [3]. Recently, D-mannose supplements have been reported to apply in treating inflammatory skin diseases, including atopic dermatitis [25] and psoriasis [38, 39]. We previously found that D-mannose achieved great efficacy in skin disease treatment without obvious adverse effects occurrence [25]. Therefore, we were intrigued to explore the potential topical application of D-mannose for preventing the deterioration of nonhealing wounds in diabetics. In this present study, we first demonstrated the protective effect of D-mannose on wound closure in STZ-induced diabetic mice and high-glucose stimulated keratinocytes. Our results first showed that skin topical supplementation of D-mannose facilitated the wound healing process by inhibiting AGEs production and therefore activating AMPK/Nrf2/HO1 to maintain physiological functions in keratinocytes. Overall, our findings did emphasize the protective role of D-mannose in treating diabetic wounds, especially through the topical administration route.

In present, the treatment and prevention of diabetic wound is an insurmountable issue in clinical practice due to the lack of safe and effective therapy [6]. The wounds in patients suffering from diabetes fail to repair due to the hyperglycemia and chronic inflammation. Since D-mannose is not only anti-inflammatory with a biosafety profile but also effective via skin topical administration as we previously reported [25], we here topically supplemented D-mannose on mice diabetic wounds. We surprisingly found that the topical use of D-mannose is an effective route of administration, greatly accelerating wound healing process and decreasing complete closure time in STZ-induced diabetic mice without the occurrence of adverse effects. Interestingly, D-mannose treatment did not exhibit significant advantages of wound healing in mice without diabetes, indicating that D-mannose plays its beneficial role under hyperglcemia condition. Recent studies have proved that long-term high blood sugar environment is responsible for wound unhealing and other diabetes complications via the accumulation of excessive AGEs [40, 41]. In our current investigation, D-mannose greatly inhibited the level of AGEs in diabetic mice lesional skin as well as in high glucose treated keratinocytes. AGEs are a series of stable compounds generated by glycation reaction between glucose and free amino groups in proteins, nucleic acids and lipids [10]. D-mannose has been reported to disturb intracellular glucose metabolism and downstream biological functions [30, 42]. Nevertheless, the present study found that blood glucose concentration does not change after the topical treatment of D-mannose, which is in line with clinical results of D-mannose supplement in diabetics without altering glucose levels [43]. Furthermore, D-mannose could not protect cells from apoptosis induced by exogenous AGEs addition. Together, our current findings proved that the topical supplementation of D-mannose promoted diabetic wound closure mainly targeting AGEs production, but not the decrease of blood sugar or curb of AGEs function.

Accumulation of AGEs in skin leads to dysfunction of skin barrier, excessive inflammation and degraded extracellular matrix [10]. Considering that AGEs are enriched in epidermis [44] where keratinocyte accounts for more than 90% [45], we then explored the underlying mechanism of D-mannose mainly focusing on keratinocytes. Here, we found that the normal physiologic functions of keratinocytes (including, cell viability, migration, differentiation and non-inflammation) are greatly compromised with the high glucose stimulation mimicking in vivo hyperglycemia, while D-mannose treatment markedly protected and maintained physiologic ability in keratinocytes. It is worthy to mention that AMPK signaling is a pivotal effect target for D-mannose [33]. Furthermore, AMPK/Nrf2/HO-1 pathway is revealed as a classic pathway during the pathogenesis of diabetic-associated diseases, especially diabetic wounds [46, 47]. For these above reasons, we wondered whether D-mannose could inhibit AGEs production to regulate AMPK pathway, ultimately rescuing dysfunctional keratinocytes in hyperglycemic environment. As expected, D-mannose indeed upregulated AMPK/Nrf2/HO-1 signaling both in vivo and in vitro. Accordingly, the protective effect of D-mannose was abolished upon pretreatment with AMPK or AGEs inhibitor, confirming that suppression of AGEs formation and activation of downstream AMPK/Nrf2/HO-1 are responsible for the effect of D-mannose on regulating normal functional keratinocytes under high glucose circumstances. The experimental results in our current study are supported by a previous report found that excessive AGEs could markedly suppress AMPK/Nrf2/HO-1 and subsequently induce cell death or malfunction [36, 48]. Numbers of evidence supported that, inflammatory keratinocyte induced by hyperglycemia not merely exhibits dysfunction but also negatively influences other neighbouring cell populations via cytokines within the diabetic wound microenvironment [17, 49, 50]. Notedly, we further found that D-mannose treated keratinocytes could suppress proinflammatory M1 macrophage polarization and support fibroblast growth, which was also confirmed by in vivo results. On the whole, D-mannose reduced AGEs formation to sustain keratinocyte physiological function, orchestrating multiple cell populations to promote diabetic wound healing.

## Conclusions

In summary, this study first unveils the beneficial role of topical supplementation of D-mannose on wound healing in the context of diabetes. Moreover, D-mannose could impair the formation of AGEs, thus preserving the physiologic condition of keratinocytes via AMPK/Nrf2/HO-1 signaling pathway upregulation in the context of hyperglycemia (Fig. 6F). To sum up, our present results suggest that D-mannose is a promising skin-topical-applied therapy in treating diabetic wound healing, and further expanding the clinical application range of D-mannose.

## Electronic supplementary material

Below is the link to the electronic supplementary material.

Supplementary Material 1

## Data Availability

No datasets were generated or analysed during the current study.

## Abbreviations

DMEM: Dulbecco’s modified Eagle’s medium
PBS: Phosphate buffered saline
STZ: Streptozotocin
AGEs: Advanced glycation end products
ROS: Reactive oxygen species
TNF-α: Tumor necrosis factor-α
IL-8: Interleukin-8
