# Supplementary material for: D-mannose promotes diabetic wound healing through inhibiting advanced glycation end products formation in keratinocytes
Source: Mol Med. 2025 Jan 18;31:15. doi: 10.1186/s10020-025-01070-3 (PMC11748336; doi:10.1186/s10020-025-01070-3)
Supplement: Supplementary file 1 — Supplementary Material 1 [file 10020_2025_1070_MOESM1_ESM.docx]

**Table 1 Primers for Mice**

|  | Forward primer (5’-3’) | Reverse primer (5’-3’) |
| --- | --- | --- |
| *Involucrin* | ATGTCCCATCAACACACACTG | TGGAGTTGGTTGCTTTGCTTG |
| *Loricrin* | GCGGATCGTCCCAACAGTATC | TGAGAGGAGTAATAGCCCCCT |
| *Filaggrin* | ATGTCCGCTCTCCTGGAAAG | TGGATTCTTCAAGACTGCCTGTA |
| *β-actin* | GGCTGTATTCCCCTCCATCG | CCAGTTGGTAACAATGCCATGT |

**Table 2 Primers for human**

|  | Forward primer (5’-3’) | Reverse primer (5’-3’) |
| --- | --- | --- |
| *TNF-α* | CCTCTCTCTAATCAGCCCTCTG | GAGGACCTGGGAGTAGATGAG |
| *IL-8* | AAATTTGGGGTGGAAAGGTT | AAGAAACCACCGGAAGGAAC |
| *Involucrin* | TCCTCCAGTCAATACCCATCAG | CAGCAGTCATGTGCTTTTCCT |
| *Loricrin* | GAGGTGTTTTCCAGGGGCA | TGGGGTTGGGAGGTAGTTGTA |
| *Filaggrin* | TGAAGCCTATGACACCACTGA | TCCCCTACGCTTTCTTGTCCT |
| *α-SMA* | CTATGAGGGCTATGCCTTGCC | GCTCAGCAGTAGTAACGAAGGA |
| *Collagen I* | CCGGCTCCTGCTCCTCTTAGCG | CGTTCTGTACGCAGGTGATTGGTGG |
| *Fibronectin-1* | AGGAAGCCGAGGTTTTAACTG | AGGACGCTCATAAGTGTCACC |
| *β-actin* | CACCATTGGCAATGAGCGGTTC | AGGTCTTTGCGGATGTCCACGT |
